# Characteristics of N400 component elicited in patients who have migraine with aura

**DOI:** 10.1186/s10194-021-01375-8

**Published:** 2021-12-27

**Authors:** Igor Petrusic, Vojislav Jovanovic, Vanja Kovic, Andrej Savic

**Affiliations:** 1 Laboratory for Advanced Analysis of Neuroimages, Faculty of Physical Chemistry, University of Belgrade, 12-16 Studentski Trg Street, Belgrade, 11000 Serbia; 2 Laboratory for Neurocognition and Applied Cognition, Department of Psychology, Faculty of Philosophy, University of Belgrade, Belgrade, Serbia; 3 Science and Research Centre, School of Electrical Engineering, University of Belgrade, Belgrade, Serbia

**Keywords:** Electroencephalography, Event-related potentials, Headache, Semantic processing, Source localization

## Abstract

**Background:**

This study aimed to examine the N400 effect and event-related potentials (ERPs) elicited from congruent and incongruent stimuli in patients who have migraines with aura (MwA).

**Methods:**

A total of 33 MwA patients and 20 healthy controls (HCs) were studied. They were balanced in age (35.12 ± 8.94 vs 34.70 ± 9.59 years, *p* = 0.872) and sex (69.7 vs 75.0% females, *p* = 0.761). ERPs were measured in response to both stimuli, where pictures were preceded with an object name that either matched or mismatched with the object. Averaged amplitudes, peaks, peak latencies, difference waves and topography were compared between MwA and HCs.

**Results:**

MwA patients had significantly lower averaged amplitudes at the Fz and F4 sites during incongruent stimuli, as well as reduced peaks at the C3 and Pz sites. Topography showed a more widespread N400 effect over scalp relative to HCs. The difference ERP waveforms did not differ in the N400 effect between groups, but the P600 effect was significantly stronger in the HCs group relative to the MwA group at the Pz (6.52 ± 2.57 vs. 3.50 ± 3.15, *p* = 0.001) and P4 (5.86 ± 2.79 vs. 3.95 ± 3.64, *p* = 0.040) sites.

**Conclusions:**

Picture-word matching tasks could serve as a potential new method for the investigation of semantic processing in MwA patients.

## Introduction

Migraine with aura (MwA) is a worldwide highly prevalent disorder that can have a tremendous impact on everyday life [1, 2]. Although it is well known that during MwA attack cognitive dysfunction is present in various forms, recent studies also suggest that MwA patients could suffer from subtle cognitive changes during the interictal period [3–6]. Furthermore, it is known that cortical spreading depression, which is a pathophysiological substrate of migraine aura, could disrupt cortical function and also could be a cumulative risk factor for cerebrovascular events [7].

All of the above support the importance of investigating the cortical dysfunction outside of MwA attack as a relevant target for further better understanding of complex and multilayered MwA pathophysiology. Moreover, given that dysphasia is a common symptom during MwA attack [8] and that MwA patients also can have subtle impairment of verbal functioning interictally [9, 10], new approaches to the investigation of interictal verbal functioning and cognitive function in MwA patients are needed.

One of the interesting ways to investigate subtle semantic differences changes is an N400 component of the event-related potential (ERP) measured by electroencephalography (EEG) [11]. N400 component could be observed in lexical priming paradigms, where a target word was or was not somehow related to an immediately preceding (prime) word or picture. Compared to a congruent condition where words and object referents are presented correctly with one another, words and pictures that are not matching elicit an ERP component that has a more negative amplitude, peaking around 300–500 milliseconds (ms) after the onset of the word, with a most visible effect on centro-parietal electrodes [12]. An advantage of this technique is that it provides an immediate and continuous record of the neural processes associated with evaluating a cognitive stimulus with a high temporal resolution, allowing to directly and precisely measure when different computational processes underlying semantic memory are taking place in the brain [11, 13].

There are no papers, to our knowledge, that explored brain activation in a picture-word priming paradigm using event-related potentials during an interictal period in MwA patients. This study aims to correct this lack by studying ERP parameters in a group of patients who have episodic migraines with typical aura and to compare them with healthy controls (HCs). Since ERPs are already recognized as promising biomarkers which can provide sensitive, objective and reliable measures of the neural events underlying cognition in neurological disorders [14], this study aims to pave the way for exploring their potential role as biomarkers in migraine. Moreover, ERPs-based biomarkers could be able to identify different MwA phenotypes or serve as a measure of the response to specific treatment [15].

## Methods

### Participants

Thirty-five patients with exclusively episodic typical MwA, according to the International Headache Society criteria [16], were recruited between 2019 and 2020 from the migraine population referring to the Center for headaches, Neurology Clinic, Clinical Center of Serbia. Patients were without neurological (other than MwA), psychiatric, cardiovascular and metabolic disorders. All patients were both migraine-free and not taking any medications at least 3 days before electrophysiological recordings of a brain during a cognitive task. Also, all patients did not take any migraine preventive medications at the time of the study. Additionally, twenty-three age- and sex-balanced HCs with no family history of migraine or other neurological and other chronic systemic diseases were recruited. HCs were voluntarily recruited from clinical staff or their relatives and friends, who upon acceptance underwent physical and neurological examinations. Also, MRI was performed to exclude intracranial lesions in all participants.

The study was approved by the Scientific Ethics Committee of Clinical Center of Serbia and Neurology Clinic (reference number: 23–690). The study conforms with the World Medical Association Declaration of Helsinki. The subjects signed a written informed consent form before participation.

### ERP study design and processing of signals

**Fig. 1:**
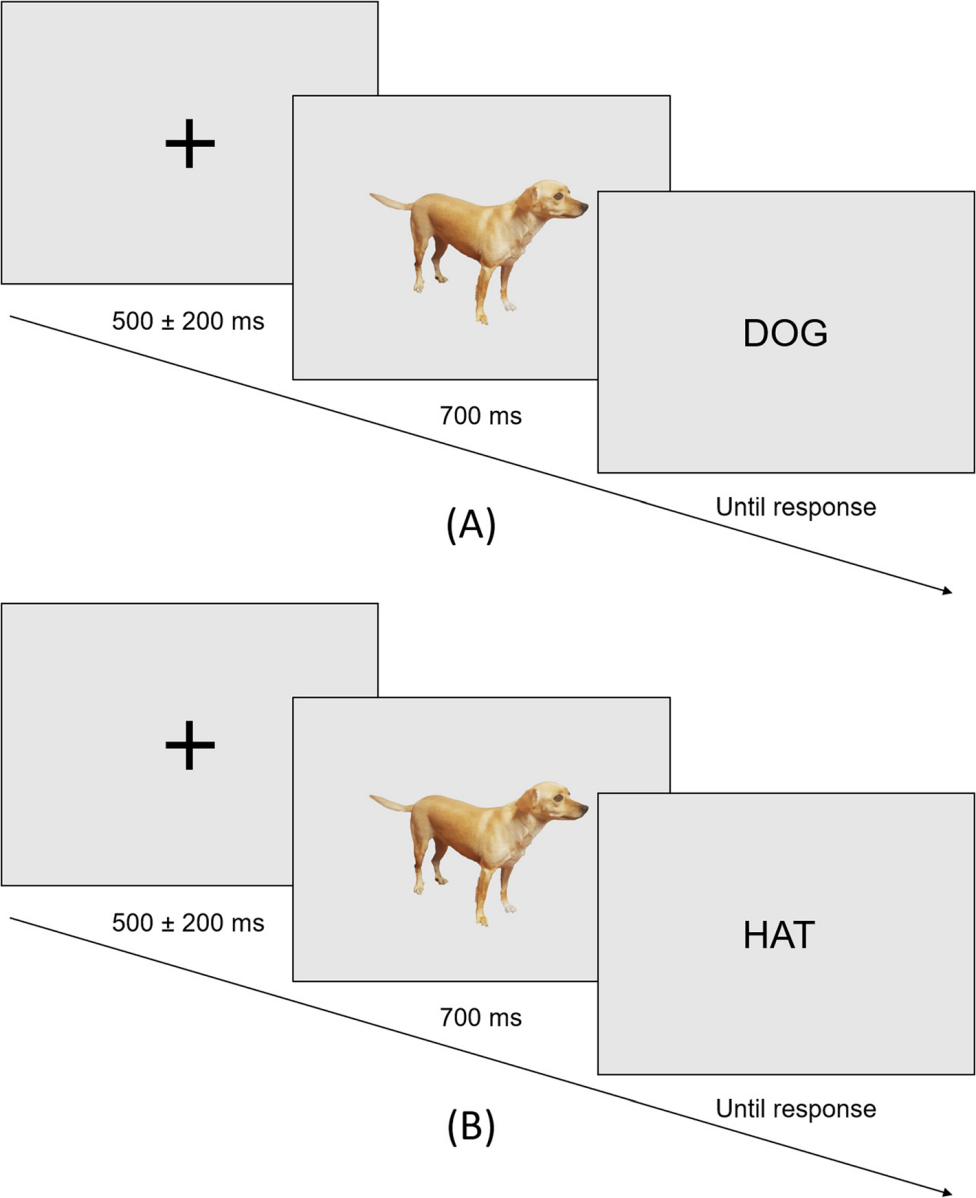
Examples of experimental trials for a picture-word match (**A**) and mismatch (**B**) conditions

Participants were asked to sit in a comfortable chair in an electrically shielded room and to observe a 17-in CRT monitor that was placed 60 cm in front of them. Each trial started with a fixation cross in the center of the screen for the jittered time range between 300 and 700 ms that varied from trial to trial. Next, the image appeared for 700 ms, immediately followed by the target word which remained on the screen for three seconds or until response (Fig. 1). Participants were instructed to press the left mouse button click for the picture-word match (congruent stimulus) and the right mouse button for the picture-word mismatch (incongruent stimulus). Words appeared in black Mono 24 px font against a light gray background. There were in total 120 trials including 60 trials for match condition and 60 trials for mismatch condition. For stimuli presentation, we used OpenSesame 3.3.9 [17].

Stimuli were obtained by using image databases Photodisc collection and Hemera Photo-Objects, as well as internet Google Image Search. Sixty pictures of easily nameable and recognizable objects, such as animals, everyday objects, fruits, etc. were selected. All stimuli were cropped and resized to fit a box of approximately 300 × 300 pixels, presented on a light gray background in the middle of the screen (to prevent eye movements during the task). Each image was paired with a target word (noun), which was related (name of the picture) or unrelated to the presented picture, making two picture-word sets (match vs. mismatch condition).

EEG signals were recorded continuously from the scalp in monopolar setup from 35 electrode sites positioned according to the international 10/20 standard: Fp1, Fp2, F7, F8, FT9, FT10, T7, T8, F3, Fz, F4, FC5, FC6, FC1, FC2, FCz, C3, Cz, C4, CP5, CP6, CP1, CP2, P3, Pz, P4, TP9, TP10, P7, P8, PO9, PO10, O1, Oz, and O2. All electrodes were referenced to the left earlobe reference, and the ground electrode was positioned at the AFz location. Skin-electrode contact impedance levels were maintained below 5 kO. EEG was recorded with a sampling rate of 1000 Hz.

Offline signal processing was conducted using custom MATLAB routines (version 2015a, The Mathworks, Natick, MA, U.S.A.). All EEG channels were band-pass filtered using a zero-phase 4th order Butterworth filter with 0.1–25 Hz cut-off frequencies. Individual 1000 ms EEG epochs, with 0 marking the stimulus, which included 100 ms pre-stimulus baseline and 900 ms post-stimulus data, were extracted from the continuous filtered EEG. All EEG channels were baseline corrected by subtracting the mean amplitude of the 100 ms baseline from each epoch. The trials were inspected for artifacts and only the noise-free trials were included in further analyses. Data from 2 MwA patients and 3 HCs were rejected due to the presence of noise which resulted in a high number of rejected epochs per experimental condition (> 30). For each participant and each condition at each electrode site, individual ERPs were calculated by averaging all remaining trials. Additionally, the difference ERP waveforms were calculated for each subject by subtracting the average ERPs (of each channel) of the incongruent condition from the averaged ERPs of congruent condition.

Average ERPs and difference waves for each channel were segmented into 20 ms non-overlapping time bins and mean amplitude for each time bin was calculated.

### Statistical analyses

For the analyses of demographic and clinical variables among groups, we used descriptive statistics (mean ± standard deviation and percentage), parametric test (the Independent Student T-test for age) and nonparametric test (the Chi-square test for sex). *P* < 0.05 was considered statistically significant.

Effects of MwA on priming were assessed with an analysis of variance with grouping factors of participant status (MwA vs. HCs), experimental condition (congruent vs. incongruent stimuli) and recording site. The recording site included two dimensions: anterior-posterior distribution and laterality. The anterior-posterior dimension grouped frontal (F3, Fz, F4), central (C3, Cz, C4), and parietal (P3, Pz, P4) electrodes. The laterality dimension grouped left (F3, C3, T3), middle (Fz, Cz, Pz), and right (F4, C4, P4) electrodes. The average amplitude value of the selected window (260–460 ms) for the determined N400 component was used for mixed model repeated ANCOVA which was corrected for sex and age of participants. We applied mean amplitude measurement that calculates the mean voltage of the waveform in a predetermined window of time because this method is advised when investigated cognitive processes do not occur at fixed latencies over trials or subjects [18]. In the case of significant interactions, they were broken down following subsequent analysis in an attempt to understand the locus of the interaction. Significant main effects were further explored by follow-up t-tests. A Greenhouse-Geisser correction was used in cases of sphericity violation. The *p*-value for significance testing was 0.05. For the analysis of amplitudes between groups, we used the Mann-Whitney U test, while the Independent Student T-test was used for the analysis of latency differences. The false discovery rate (FDR) correction for multiple comparisons was applied. Also, statistical analysis of peaks and latencies was conducted for a selected window at all pre-selected channels.

Furthermore, for deeper exploratory analysis, we aimed to identify for each of the selected subsets of 9 EEG channels electrodes around a vertex (F3, Fz, F4, C3, Cz, C4, P3, Pz, P4) the time instants corresponding to statistically significant differences between groups. The Mann-Whitney U test and Independent t-test were used for this purpose.

## Results

A total of 33 MwA patients and 20 HCs were studied. They were balanced in age (35.12 ± 8.94 vs 34.70 ± 9.59 years, *p* = 0.872) and sex (69.7 vs 75.0% females, *p* = 0.761). The average disease duration was 17.81 ± 11.02 years. MwA attack frequency per year was 6.00 ± 6.55.

**Fig. 2:**
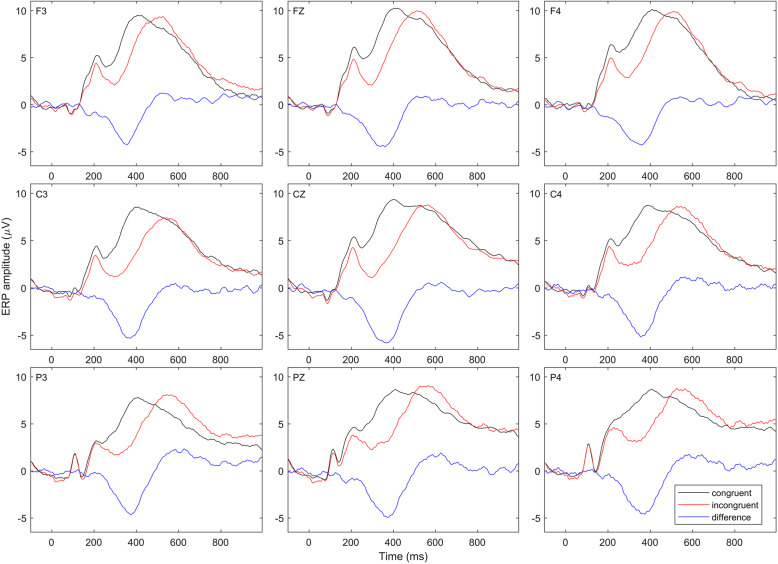
The grand averaged ERP curves of congruent (black line) and incongruent condition (red line) were presented at the frontal, central and posterior channels. The blue line represents the difference wave which reveals the N400 effect

The grand averaged ERP curves of both experimental conditions, including difference wave, for all participants at the frontal (F3, Fz, F4), central (C3, Cz, C4) and posterior (P3, Pz, P4) regions are shown in Fig. 2. The window (260–460 ms) that shows the N400 effect was used for the calculation of averaged amplitude value for each participant and repeated ANCOVA was conducted. Mixed model repeated measures ANCOVA showed the main effect of experimental condition (*p* < 0.001), with lower amplitudes in mismatch condition (incongruent stimuli). The main effect of the region was also detected (*p* = 0.037), with lower amplitudes on central and posterior electrodes compared to the frontal set (*p* < 0.001). There was no significant interaction between effect and groups (*p* = 0.200). A region x laterality x group interaction was also detected (*p*  = 0.028), as well as region x group interaction (*p* = 0.007). The age (*p* = 0.538) and sex (*p* = 0.331) of participants did not influence significance of the results. Because of the complex scalp distribution effects, follow-up tests were conducted at each of the preselected sites to examine detected interactions.

Comparison between groups relative to the experimental condition, N400 component and electrode sites revealed that during incongruent stimuli MwA patients had significantly lower amplitudes at the Fz and F4 sites (Table 1). Also, incongruent stimuli pointed to the difference between groups at the C3, Cz, C4 and Pz sites, but statistical significance did not persist after the FDR correction. The peak of the N400 component during the incongruent stimuli was significantly lower in MwA patients relative to HCs at the C3 and Pz sites. Moreover, incongruent stimuli pointed to the difference between groups at the Fz, F4, Cz, C4, P3 and P4 sites, but statistical significance did not persist after the FDR correction. The peak latency of the N400 component during the incongruent stimuli was reduced in MwA patients relative to HCs at the Pz and P4 sites.

**Table 1:** Amplitudes and latencies derived from 9 pre-selected channels for the congruent and incongruent conditions in MwA and HCs

		Congruent condition			Incongruent condition		
		MwA (*n* = 33)	HCs (*n* = 20)	Statistics	MwA (*n* = 33)	HCs (*n* = 20)	Statistics
F3	Averaged N400 (μV)	6.20 ± 3.55	9.50 ± 6.47	*p* = 0.095	3.51 ± 3.94	7.13 ± 6.55	*p* = 0.072
	Peak (μV)	10.85 ± 5.70	15.01 ± 9.65	*p* = 0.132	8.65 ± 5.21	13.18 ± 9.97	*p* = 0.095
	Latency (ms)	380 ± 60	389 ± 49	*p* = 0.600	396 ± 71	393 ± 70	*p* = 0.876
Fz	Averaged N400 (μV)	6.61 ± 4.28	10.88 ± 7.37	*p* = 0.099	2.95 ± 4.39	8.28 ± 6.31	***p*** = 0.004*
	Peak (μV)	10.89 ± 5.43	16.82 ± 10.46	*p* = 0.056	8.01 ± 5.16	14.57 ± 9.35	***p*** **= 0.010**
	Latency (ms)	383 ± 62	387 ± 59	*p* = 0.818	397 ± 72	422 ± 51	*p* = 0.145
F4	Averaged N400 (μV)	7.10 ± 3.58	10.52 ± 6.94	*p* = 0.110	3.55 ± 3.79	8.20 ± 6.14	***p*** = 0.004*
	Peak (μV)	10.96 ± 5.19	16.30 ± 10.50	*p* = 0.078	8.27 ± 4.94	14.28 ± 10.27	***p*** **= 0.024**
	Latency (ms)	383 ± 58	393 ± 58	*p* = 0.576	407 ± 74	419 ± 54	*p* = 0.538
C3	Averaged N400 (μV)	5.67 ± 3.21	8.23 ± 4.90	*p* = 0.085	1.55 ± 3.59	4.88 ± 4.20	***p*** **= 0.009**
	Peak (μV)	9.42 ± 4.03	12.60 ± 6.91	*p* = 0.097	5.44 ± 4.23	9.93 ± 6.35	***p*** = 0.004*
	Latency (ms)	392 ± 52	388 ± 50	*p* = 0.751	404 ± 70	402 ± 72	*p* = 0.915
Cz	Averaged N400 (μV)	6.46 ± 3.66	9.49 ± 5.64	***p*** **= 0.036**	1.82 ± 4.22	5.17 ± 4.74	***p*** **= 0.024**
	Peak (μV)	10.25 ± 4.16	14.16 ± 7.45	*p* = 0.072	5.98 ± 4.90	10.53 ± 6.60	***p*** **= 0.009**
	Latency (ms)	388 ± 51	388 ± 55	*p* = 0.961	397 ± 75	402 ± 74	*p* = 0.808
C4	Averaged N400 (μV)	6.64 ± 3.03	8.55 ± 5.70	*p* = 0.193	2.56 ± 3.61	5.75 ± 4.56	***p*** **= 0.017**
	Peak (μV)	9.93 ± 3.61	13.02 ± 7.17	*p* = 0.119	6.60 ± 4.35	10.57 ± 6.46	***p*** **= 0.020**
	Latency (ms)	371 ± 58	383 ± 51	*p* = 0.444	395 ± 78	402 ± 75	*p* = 0.757
P3	Averaged N400 (μV)	5.52 ± 3.02	7.11 ± 4.41	*p* = 0.279	2.28 ± 3.38	4.33 ± 3.65	*p* = 0.075
	Peak (μV)	8.72 ± 3.16	11.13 ± 5.13	*p* = 0.102	5.81 ± 4.45	9.19 ± 4.08	***p*** **= 0.007**
	Latency (ms)	390 ± 48	383 ± 57	*p* = 0.616	385 ± 76	411 ± 71	*p* = 0.228
Pz	Averaged N400 (μV)	6.75 ± 3.13	7.89 ± 5.03	*p* = 0.769	2.74 ± 3.15	5.13 ± 3.71	***p*** **= 0.016**
	Peak (μV)	10.04 ± 2.96	12.52 ± 5.29	*p* = 0.128	6.23 ± 3.77	10.17 ± 4.66	***p*** = 0.001*
	Latency (ms)	385 ± 55	375 ± 62	*p* = 0.554	358 ± 84	409 ± 74	***p*** **= 0.030**
P4	Averaged N400 (μV)	7.39 ± 3.06	7.72 ± 4.91	*p* = 0.927	3.68 ± 2.87	4.86 ± 3.72	*p* = 0.248
	Peak (μV)	10.32 ± 3.24	11.40 ± 5.35	*p* = 0.533	7.13 ± 3.10	9.49 ± 4.21	***p*** **= 0.021**
	Latency (ms)	374 ± 59	384 ± 60	*p* = 0.574	336 ± 83	386 ± 92	***p*** **= 0.049**

*MwA* patients who have migraine with aura, *HCs* healthy controls, *μV* microvolts, *ms* milliseconds, *Averaged N400* averaged amplitudes from N400 effect window (260–460 ms), * – significant after FDR correction

**Fig. 3:**
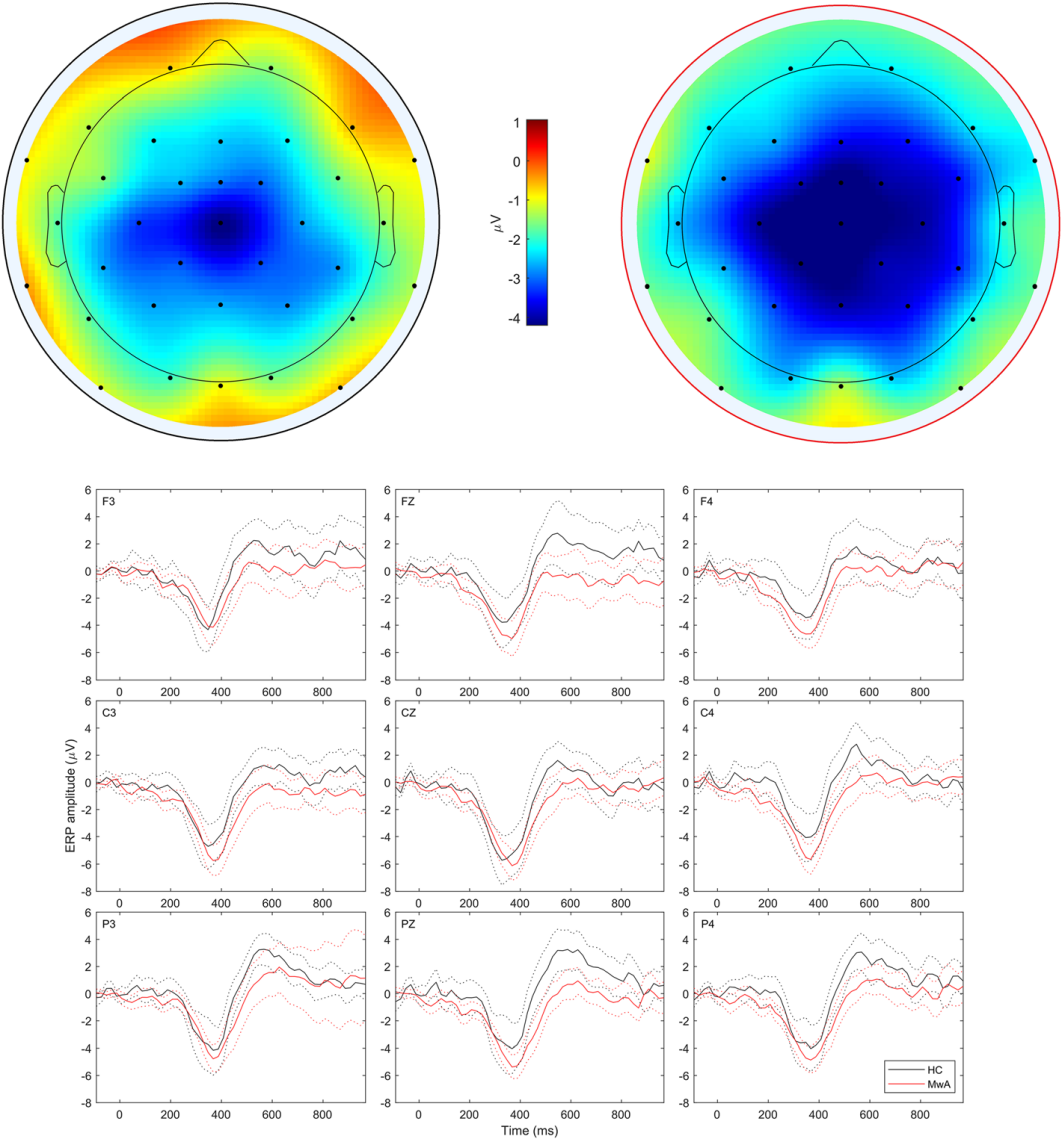
The difference waves of N400 effect and topography maps derived from the grand averaged ERPs elicited by task stimuli in MwA and HCs. The N400 component (topographical plot of mean amplitude in 260–460 ms window) is widely distributed over frontal, central, and parietal areas with the center at Cz in MwA patients (right topography), whereas in the HCs group (left topography) N400 component is distributed over central and parietal areas

Further, a comparison in terms of the N400 effect revealed that topography differs between groups (Fig. 3), showing that the N400 effect in the HCs group is localized around the Cz site and in the MwA group is more widespread over central sites with extension towards posterior and frontal sites. Also, differences are noticed on the averaged difference ERP waveforms, where the P600 effect was significantly stronger in the HCs group relative to the MwA group at the Pz (6.52 ± 2.57 vs. 3.50 ± 3.15, *p* = 0.001) and P4 (5.86 ± 2.79 vs. 3.95 ± 3.64, *p* = 0.040) sites. The peak latency of the N400 and P600 effects at all investigated sites did not significantly differ between these two groups.

**Fig. 4:**
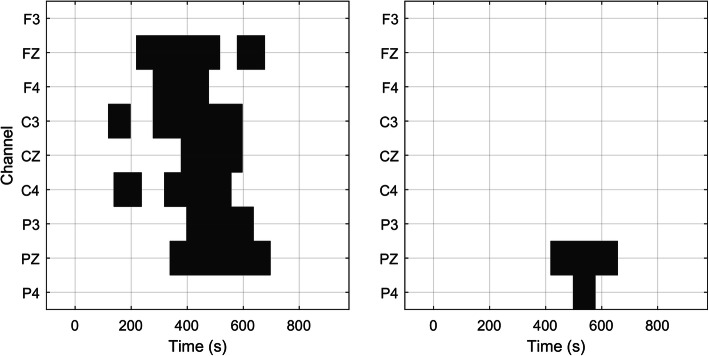
Intervals of significance between groups for incongruent stimuli (black color) and nonsignificant intervals (white color). The left graph shows the intervals for incongruent ERPs and the right graph for difference waveforms. The y-axis marks individual channels and the x-axis time points in (ms), where 0 marks the priming onset

For the purpose of deeper explorative analysis, comparisons between groups in both experimental conditions at the relevant electrodes were conducted by using averaged amplitudes of consecutive time instants. Figure 4 shows the mapping of statistical significance between groups for certain time bins. The interval of difference is considered significant only if statistically significant differences occurred in more than 3 consecutive time bins (corresponding to intervals longer than 60 ms duration) to filter out potential statistical artifacts in line with expected electrophysiological signal properties [19]. The obtained results revealed no intervals of statistical significance for the congruent condition and multiple intervals of statistical significance for the incongruent condition between groups. The earliest onset of statistical significance for the incongruent condition was observed centrally at C3 and C4 channels, starting around 120 and 140 ms, respectively. The obtained intervals of statistical significance for incongruent condition per channel are: Fz (220–520 ms and 580–680 ms), F4 (280–480 ms), C3 (120–200 ms and 280–600 ms), Cz (380–600 ms), C4 (140–240 ms and 320–540 ms), P3 (400–620 ms) and Pz (340–700 ms). Also, the results reveal two intervals of significance for difference waves at channels Pz (420–640 ms) and P4 (500–580 ms).

## Discussion

In this study, we used two experimental conditions, words that match or mismatch with presented pictures, to study semantic processing based on the N400 effect in MwA patients. The main focus was to investigate the N400 component elicited by the incongruent stimulus because there is no previous data about this effect in MwA patients, although it is known that MwA patients frequently have dysphasia during attacks [8] and certain difficulties in language processing during the interictal period were previously noted also [10]. Our analysis showed that MwA patients had responses of lower amplitudes (higher ERP negativity) to incongruent stimuli relative to HCs, although the N400 effect (quantified by difference waveforms) did not differ between examined groups.

The N400 effect was registered in a difference wave derived from incongruent and congruent ERP waves and was successfully elicited in both groups, as expected for an effect that is known to be robustly produced by all manner of semantic incongruencies [20]. The current N400 effect, in a time window between 260 and 460 milliseconds after the onset of the target, exhibited a characteristic central–parietal spatial distribution in the HCs group that resonates with previous studies conducted on healthy populations [21]. In the MwA group, there is additional distribution towards frontal regions, although there was no interaction between the N400 effect and groups, which is rather expected because violation of this effect, for cross-modal picture-word matching tasks, is present only in a severe form of mental diseases, such as schizophrenia [22]. A region x laterality x group interaction analysis showed that the difference between groups was strongest in the central and right-frontal regions, while in other regions there was a trend towards the same pattern but differences did not reach significance, although it is quite noticeable on the topography for MwA patients (Fig. 3). This was further confirmed by exploratory analysis of time intervals of statistical significance in 20 ms time bins which revealed the presence of intervals of statistical significance between groups in the central, right-frontal and left-posterior regions, for the incongruent condition. Since characterizing N400 topography across the scalp has proven difficult due to overlapping ERP components of the response to different types of stimuli [11], we could only assume that obtained different scalp topographies within the N400 window, between the groups, are the result of either a different network of cortical regions influenced by MwA pathophysiology [20] or different level of activation within the same network. Moreover, the fact that different sensory stimuli (inputs) elicit N400 systematically, but with topographic and morphological differences, implicates that the N400 component is modality dependant but not a modality-specific neural marker of processing in a distributed semantic memory system [11]. In the context of this study, the obtained morphological and topographic differences may be attributed to complex dysregulation of sensory processing in migraine. Moreover, since we have observed significant differences between the groups only for incongruent condition, our results suggest that disorder of sensory processing in MwA patients may not affect solely the low level sensory integration, but could expand to and potentially disrupt meaning construction.

Analysis of peaks and their latencies on sites of interest showed significantly lower peak amplitudes (C3 and Pz) in MwA patients during the incongruent stimuli, while latencies did not differ between groups in both experimental conditions. This pattern was visible on most of the investigated sites, although it did not reach the threshold after correction for multiple comparisons. This result can reflect, from a physiological level, smaller post-synaptic potentials and/or less temporal synchrony among the generating neurons which influence the N400 component during incongruent stimuli [11]. Combining ERP studies with functional neuroimaging techniques might reveal the real cause of these differences.

Furthermore, sophisticated analysis of the consecutive time instants confirmed our previous analyses that incongruent stimuli yield significant differences in MwA patients relative to HCs (Fig. 4). It is important to note that the congruent stimuli follow a similar pattern, although differences were not significant. Having that in mind, it can be self-explanatory why the N400 (analysis of difference waves) effect did not differ between MwA patients and HCs [23]. Moreover, these findings are similar to a study that investigated temporal lobe epilepsy and found reduced amplitude in both congruent and incongruent conditions [24]. However, these findings should be verified on other MwA populations to allow such comparisons between two pathological conditions.

From Fig. 4, it can be seen that other time windows differ between groups at a subset of selected electrodes. This finding required further analysis of ERP components where amplitude reduction during the incongruent experimental condition in MwA patients was also significant relative to HCs. More precisely, a wave that was peaking around 550–600 milliseconds showed lower peaks in the MwA group. These findings, together with lower amplitude in the N400 component, could imply that MwA patients had increased demands on semantic processing, implying possible disruption in the initial integration of visual inputs and comprehension of information associated with word processing and recognition [11]. Moreover, the so-called P600 component may reflect an additional monitoring process of semantic integration, reflecting the evaluation of whether the picture and word matching were appropriate or not [19]. P600 effect was significantly decreased in MwA patients at the posterior region, which could suggest an abnormal response to incongruent stimuli during the late phase of semantic processing as well. Knowing that MwA patients have abnormal sensitivity to environmental stimuli that can cause nonpainful discomfort [25], the P600 effect and this experimental condition could serve for future ERP studies designed to investigate MwA and their subtypes [14].

Admittedly, this study is limited by the lack of comparison with migraine patients without aura, thus our results could not be interpreted as specific for MwA patients. Given that there is a noted difference between MwA and MwoA in ERP studies that investigated the P3 component [26], we also can expect some difference between MwA and MwoA relative to the N400 component as well. Moreover, other electrophysiological techniques, such as visual and somatosensory evoked potentials, demonstrated various changes in MwA patients compared to MwoA [27], together with ERPs that elicit P3 and N400 components neurophysiological techniques could be of great help in the search for the pathophysiological basis of migraine aura. The strength of the study is the relatively large number of examined patients and sophisticated analysis of the data. Moreover, these are the first findings using the above described experimental conditions, which should establish the path for investigation of the influence of different MwA characteristics, such as headache intensity, aura manifestation, MwA frequency, depression severity and used medications, on the cognitive and semantic processing in MwA patients [6]. If any of these factors are found to be related to changes in ERPs elicited from the N400 paradigm, then the N400 component could serve as a tool for daily practice in a headache clinic as an additional measure for monitoring patients status, regarding symptoms changes in frequency and severity, follow up of the response to a specific treatment, or look for cognitive changes during MwA attack. The proposed experimental protocol is short (of 5 min average duration per subject) while the EEG measurements preparation procedure can be shortened by selecting only several EEG channels for analysis which is proven feasible in this study. This further contributes to the potential usability of this method in daily clinical diagnostic practice.

## Conclusions

Overall, the present pattern of the N400 component provided new evidence for the dysfunction of cognitive and semantic function in MwA patients during the interictal phase. Also, incongruent stimuli could serve as a potential new method for investigation of MwA pathophysiology and their consequence on cognitive and semantic processing.

## Data Availability

The datasets during and/or analysed during the current study are available from the corresponding author on reasonable request.

## Abbreviations

ERP: Event-related potentials
MwA: Migraine with aura
HC: Healthy controls
EEG: Electroencephalography
FDR: The false discovery rate
ms: Milliseconds
